# Circulating tumor DNA to monitor treatment response and detect acquired resistance in patients with metastatic melanoma

**DOI:** 10.18632/oncotarget.5788

**Published:** 2015-09-22

**Authors:** Elin S. Gray, Helen Rizos, Anna L. Reid, Suzanah C. Boyd, Michelle R. Pereira, Johnny Lo, Varsha Tembe, James Freeman, Jenny H.J. Lee, Richard A. Scolyer, Kelvin Siew, Chris Lomma, Adam Cooper, Muhammad A. Khattak, Tarek M. Meniawy, Georgina V. Long, Matteo S. Carlino, Michael Millward, Melanie Ziman

**Affiliations:** ^1^ School of Medical Sciences, Edith Cowan University, Joondalup, Western Australia, Australia; ^2^ Department of Biomedical Sciences, Faculty of Medicine and Health Sciences, Macquarie University, Sydney, New South Wales, Australia; ^3^ School of Engineering, Edith Cowan University, Joondalup, Western Australia, Australia; ^4^ Centre for Cancer Research, The University of Sydney at Westmead Millennium Institute, Westmead Hospital, Westmead, New South Wales, Australia; ^5^ Department of Medical Oncology, Crown Princess Mary Cancer Centre, Westmead Hospital, Westmead, New South Wales, Australia; ^6^ Disciplines of Pathology, The University of Sydney, Sydney, New South Wales, Australia; ^7^ Medicine, Sydney Medical School, The University of Sydney, Sydney, New South Wales, Australia; ^8^ Melanoma Institute Australia, Sydney, New South Wales, Australia; ^9^ Department of Medical Oncology, Sir Charles Gairdner Hospital, Nedlands, Western Australia, Australia; ^10^ Department of Medical Oncology, Fiona Stanley Hospital, Murdoch, Western Australia, Australia; ^11^ School of Medicine and Pharmacology, The University of Western Australia, Crawley, Western Australia, Australia; ^12^ School of Pathology and Laboratory Medicine, The University of Western Australia, Crawley, Western Australia, Australia

**Keywords:** melanoma, ctDNA, acquired resistance, MAPK inhibition, immunotherapy

## Abstract

Repeat tumor biopsies to study genomic changes during therapy are difficult, invasive and data are confounded by tumoral heterogeneity. The analysis of circulating tumor DNA (ctDNA) can provide a non-invasive approach to assess prognosis and the genetic evolution of tumors in response to therapy. Mutation-specific droplet digital PCR was used to measure plasma concentrations of oncogenic *BRAF* and *NRAS* variants in 48 patients with advanced metastatic melanoma prior to treatment with targeted therapies (vemurafenib, dabrafenib or dabrafenib/trametinib combination) or immunotherapies (ipilimumab, nivolumab or pembrolizumab). Baseline ctDNA levels were evaluated relative to treatment response and progression-free survival (PFS). Tumor-associated ctDNA was detected in the plasma of 35/48 (73%) patients prior to treatment and lower ctDNA levels at this time point were significantly associated with response to treatment and prolonged PFS, irrespective of therapy type. Levels of ctDNA decreased significantly in patients treated with MAPK inhibitors (*p* < 0.001) in accordance with response to therapy, but this was not apparent in patients receiving immunotherapies. We show that circulating *NRAS* mutations, known to confer resistance to BRAF inhibitors, were detected in 3 of 7 (43%) patients progressing on kinase inhibitor therapy. Significantly, ctDNA rebound and circulating mutant *NRAS* preceded radiological detection of progressive disease. Our data demonstrate that ctDNA is a useful biomarker of response to kinase inhibitor therapy and can be used to monitor tumor evolution and detect the early appearance of resistance effectors.

## INTRODUCTION

Most melanomas display aberrant activation of the mitogen activated protein kinase (MAPK) pathway [1], most frequently via oncogenic mutations affecting *BRAF* and *NRAS* [2]. *BRAF* mutations commonly result in the substitution of the valine at codon 600 for glutamic acid (V600E; 80%), lysine (V600K; 12%), methionine, arginine or aspartic acid (each 4-5%) [3-6]. *BRAF*^V600^-mutant melanomas are exquisitely sensitive to BRAF inhibitors such as dabrafenib and vemurafenib, which alone or in combination with a MEK inhibitor improve the overall survival of *BRAF*^V600^-mutant melanoma patients [7-10]. Despite these therapeutic advances approximately 50% of melanoma patients treated with BRAF and MEK inhibitors will progress within 12 months. Resistance usually involves MAPK reactivation, often via mutations affecting *NRAS* or *MEK*, mutant *BRAF* gene amplifications or alternative *BRAF* splicing [11-13]. Acquired resistance mechanisms differ between and within patients and also exhibit intra-tumoral heterogeneity [11, 12].

In addition to targeted therapies, recent clinical trials have demonstrated the efficacy of reactivating anti-tumor immune responses by targeting inhibitory immune receptors. Monoclonal antibodies against the CTLA-4 receptor (ipilimumab) and the PD-1 receptor (nivolumab and pembrolizumab) show remarkable long-term benefits in the 10% and 40% of patients who respond, respectively [14-17]. These immunotherapies show delayed activity, and tumor regression can occur after initial tumor enlargement [18, 19]. Altogether the above underscores the need for better prognostic markers and early indicators of response to treatment.

The analysis of circulating tumor DNA (ctDNA) can provide valuable prognostic information and reveal tumor genetic changes, including the acquisition of resistance-conferring mutations during therapy in a variety of cancers [20-24]. In melanoma, the quantity of tumor associated mutant *BRAF* ctDNA correlated with tumor burden, and lower concentrations of basal mutant *BRAF* ctDNA were associated with a higher overall response rate and longer progression-free survival (PFS) in patients treated with BRAF inhibitors [25, 26]. More recently, Lipson et al. showed that levels of ctDNA correlated with radiological outcomes in a small group of melanoma patients treated with immunotherapies [27]. Similarly Tsao et al. showed changes in ctDNA levels in six patients treated with different immunotherapy modalities that reflected changes in their disease status [28].

In this study we analysed the ctDNA in *BRAF* and *NRAS*-mutated melanoma patients at baseline (*n* = 48) and within 8 weeks of treatment initiation (*n* = 25) to determine whether ctDNA correlates with treatment response and clinical benefit. We also analysed the dynamic changes in ctDNA in response to MAPK inhibitors and immunotherapies during response and after progression. Furthermore, we evaluated the ctDNA for the presence of mutations associated with resistance to BRAF inhibitor therapy.

## RESULTS

### Baseline ctDNA levels are associated with treatment response and PFS

We quantified the amount of ctDNA in 48 patient plasma samples collected at baseline, i.e. 0-2 weeks prior to treatment initiation. ctDNA was detectable in 22 of 34 cases (65%) with *BRAF*^V600E^ tumors (one requiring a different probe as the patient carried a 1799-1800 TG > AA mutation), in 7 out of 8 patients with *BRAF*^V600K^ and in all cases with *BRAF*^V600R^ (*n* = 2) and *NRAS*^Q61K/R/L^ (*n* = 4) tumors. Detectable ctDNA levels ranged from 1.6-57,302 copies/ml. Interestingly a significant correlation was found between the concentration of ctDNA and plasma LDH activity (*n* = 26, r = 0.76, *p* < 0.0001) (Supplementary Figure 1).

**Figure 1:**
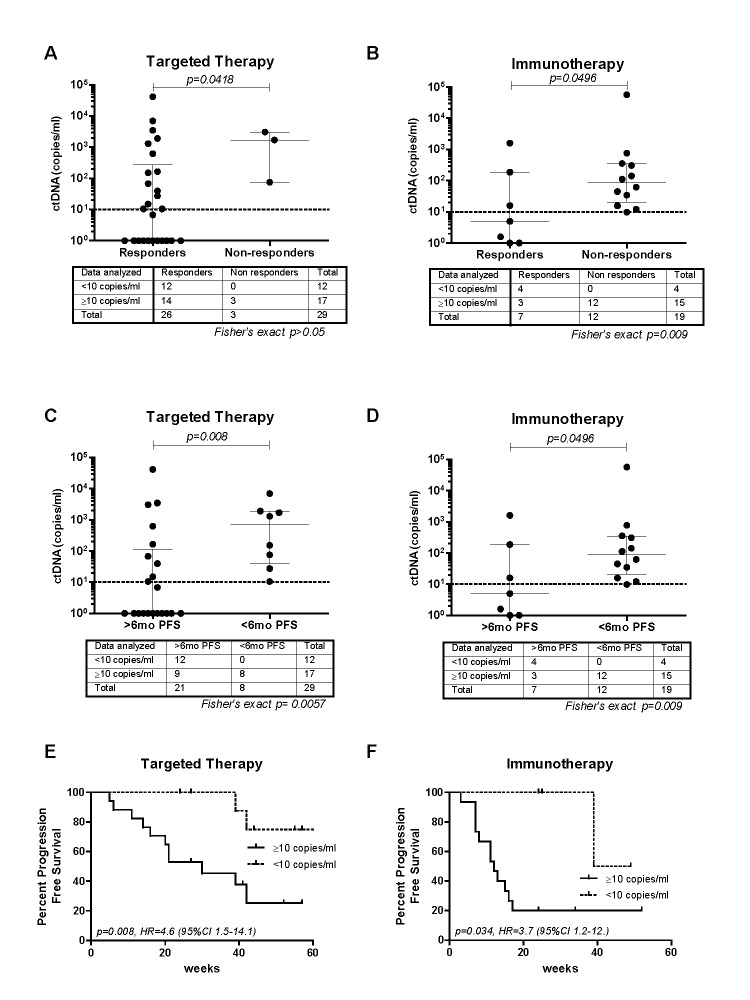
Baseline ctDNA association with response to treatment and PFS Association of baseline ctDNA concentrations with **A.** and **B.** response to treatment and **C.** and **D.** 6 months PFS. Median with interquartile range is indicated on each data set. Contingency tables with corresponding Fisher's exact test p-value are indicated below each graph. Kaplan-Meier plots of PFS probabilities according to baseline ctDNA concentrations of **E.** cases treated with targeted therapies (*n* = 29) and **F.** immunotherapies (*n* = 19). Cox regression *p*-values, Hazard ratio (HR) and confidence intervals are indicated for each plot.

Of the 48 cases analysed in this study, 29 were treated with MAPK inhibiting therapies (24 dabrafenib/trametinib, 4 vemurafenib and 1 dabrafenib monotherapy) while 19 were treated with immunotherapies (9 with ipilimumab, 3 with nivolumab, 6 with pembrolizumab and 1 with a combination of ipilimumab/pembrolizumab). Patients that responded to targeted therapy had significantly lower baseline ctDNA than non-responders (median, 10.5 versus 1695 copies/ml, *p* = 0.042, Mann-Whitney U-test) (Figure 1A). Of note, all cases with < 10 copies/ml of ctDNA at baseline (*n* = 12) responded to therapy. However this association was not statistically significant possibly due to the limited number of non-responders. Patients receiving immunotherapy that responded to treatment also had significantly lower baseline ctDNA than non-responders (median, 5 versus 87.2 copies/ml, *p* = 0.049, Mann-Whitney U-test) (Figure 1B). Moreover, baseline ctDNA (< 10 copies/ml) was significantly associated with response to immunotherapy (*p* = 0.009, Relative risk 5, 95% CI 1.8-13.8).

Similarly, targeted therapy treated patients with PFS longer than 6 months had significantly lower median baseline concentrations of ctDNA compared to those with PFS less than 6 months (1 versus 725 copies/ml, *p* = 0.008, Mann-Whitney U-test) (Figure 1C). Low baseline ctDNA levels were associated with longer than 6 month PFS (*p* = 0.0057, RR = 1.9, 95% CI 1.2-2.9). Patients receiving immunotherapy with PFS longer than 6 months also had significantly lower baseline ctDNA compared to those with less than 6 months PFS (median, 5 versus 87.2 copies/ml, *p* = 0.049, Mann-Whitney U-test) (Figure 1D). Moreover, baseline ctDNA (< 10 copies/ml) was significantly associated with 6 months PFS (*p* = 0.009, Relative risk 5, 95% CI 1.8-13.8).

In a Cox regression analysis, patients on targeted therapy with ≥10 copies/ml of ctDNA at baseline had a significantly shorter PFS compared to patients that had < 10 copies/ml of ctDNA (*p* = 0.008, Hazard ratio = 4.6, 95% CI, 1.5-14.1) (Figure 1E). A multivariate Cox regression analysis revealed that the predictive value of low baseline ctDNA on longer PFS in patients treated with MAPK inhibitors remained significant (*p* = 0.027) and independent of sex, age, tumor stage or LDH levels (Table 1). Low ctDNA levels (< 10 copies/ml) were also statistically significant associated with longer PFS for patients treated with immunotherapies (*p* = 0.034, Hazard ratio = 3.7, 95% CI 1.2-12.5) (Figure 1F).

**Table 1:** Factors associated with PFS in patients treated with targeted therapies

Factor	Variable	Univariate analysis*			Multivariate analysis*		
		p-value	HR	CI (95%)	p-value	HR	CI (95%)
ctDNA	*<10 or >=10 copies/ml*	***0.017***	***6.34***	***1.38-29.12***	***0.027***	***5.79***	***1.22-27.4***
Age	*Continuous*	***0.046***	***0.97***	***0.93-0.99***	*0.248*	*0.97*	*0.93-1.02*
Sex	*Male vs. Female*	*0.392*	*0.78*	*0.43-1.39*	*0.548*	*0.81*	*0.42-1.59*
LDH	*Elevated vs. Not elevated*	*0.848*	*1.16*	*0.26-5.27*	*0.704*	*0.72*	*0.14-3.81*
Stage	*M1a,b vs. M1c*	*0.356*	*0.62*	*0.22-1.72*	*0.741*	*0.82*	*0.26-2.59*

**Univariate and multivariate Cox regression analysis**

Overall these results suggest that low baseline ctDNA is a good predictor of response to treatment and longer PFS. Notably, of the 32 cases with high baseline ctDNA levels, 17 responded to therapy and 12 had a PFS longer than 6 months, suggesting that low baseline ctDNA (< 10 copies/ml) is predictive, but not an absolute indicator of treatment outcome and clinical benefit.

### Decrease in ctDNA after therapy initiation

**Figure 2:**
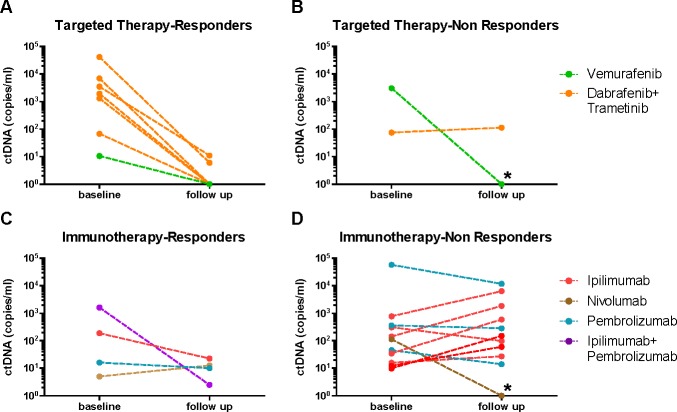
Change in ctDNA levels after therapy initiation relative to baseline Plasma samples were tested for ctDNA at baseline and between 4 to 8 weeks after therapy initiation (follow up). Cases are colour coded according to therapy, and grouped by therapy type and response. * indicates two patients that were classified as non-responders but had stable disease for at least 6 months.

A subgroup of 25 patients (10 on MAPKi and 15 on immunotherapy) with detectable ctDNA at baseline was also sampled between 4 to 8 weeks after therapy initiation. The concentration of plasma ctDNA significantly decreased in most patients after therapy initiation compared to baseline concentrations. This decrease (which ranged from 100-1000 fold reduction) was more apparent amongst patients treated with and responding to MAPK inhibitors (*p* = 0.0071) (Figure 2A). By contrast, there was no apparent decrease in ctDNA, within a similar time frame, in most patients receiving immunotherapies (Figure 2C and 2D). Of note, only 4 of the 15 immunotherapy-treated patients analysed in this subgroup, responded to treatment (Figure 2C). A 10-fold reduction in ctDNA was observed in 2 of these 4 responders, while the other 2 had a low baseline ctDNA concentration, and this remained low, but detectable, 6 weeks later.

Two patients (one treated with vemurafenib and one with nivolumab) defined as non-responders, also had a 10-fold reduction in ctDNA levels (marked with asterisks in Figure 2B and 2D). Both of them had prolonged stable disease for greater than 6 months, suggesting that the treatment had some effect on tumor activity, reflected in the decreasing ctDNA levels. One of these patients was treated with nivolumab and had evidence of progressive disease at first scan. The patient received second modality treatment with radiotherapy to an enlarging chest wall lesion and continued treatment with anti-PD-1 antibody. He achieved ongoing disease control for greater than 6 months. This atypical pattern of response has been described in 4% of patients in the early nivolumab trials, where subsequent response is observed after having progressive disease (PD) on initial assessment [29, 30].

### Changes in ctDNA levels in melanoma patients during therapy and at progression

**Figure 3:**
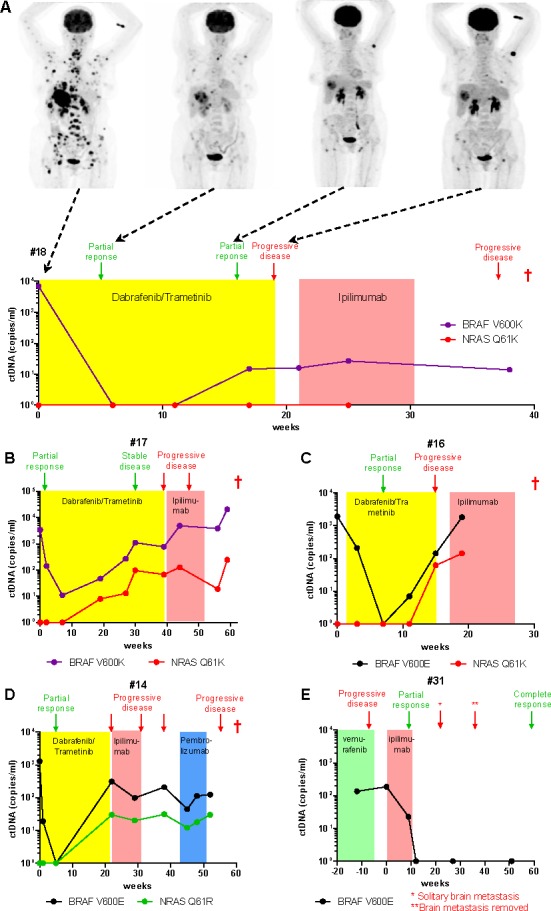
Monitoring ctDNA in plasma during clinical disease course Levels of *BRAF* and *NRAS* mutated ctDNA in plasma collected longitudinally from five melanoma patients during treatment with **A.**-**D.** dabrafenib and trametinib combination therapy, followed by immunotherapies and **E.** during ipilimumab treatment. Clinical outcomes revealed by CT and/or PET scans are indicated at each assessment time (arrows). Patient death is indicated by a red cross (†). **A.** PET scan images at four clinical assessment time points for comparison with ctDNA levels.

We monitored ctDNA level in four patients, treated with dabrafenib/trametinib as first line therapy (Figure 3A-3D). In all four patients a dramatic decline in ctDNA concentrations was recorded within 1-3 weeks after commencing treatment, decreasing to undetectable levels in 3 patients by 6 weeks. However, ctDNA rebounded in all cases prior to, or at the time of, detection of progressive disease by CT scans. In particular, in patient #17 (Figure 3B) a clear recurrence of tumor burden was apparent by ctDNA analysis at 30 weeks after treatment initiation, however the radiological analysis classified the disease status as stable at that time with progression only noted at 39 weeks. All four patients were treated with ipilimumab after failing the MAPK inhibitor therapy, but none responded to immunotherapy. The presence of ctDNA remained detectable during and after immunotherapy.

Of note, a patient that experienced complete response to treatment (Figure 3E), developed a solitary brain metastasis of 18 millimetres at week 22, but ctDNA rebound was not detected in plasma sampled prior to stereotactic radiosurgery at week 27. This observation may question the utility of ctDNA quantification in the case of brain metastases, consistent with previous studies suggesting that the blood-brain barrier may prevent ctDNA from entering the circulation [20].

### Detection of acquired resistance mutations in ctDNA

Mutations in *NRAS* have been found in 8-26% of patients with acquired resistance to BRAF inhibitors [11, 13]. We analysed the presence of *NRAS*^Q61K^ and *NRAS*^Q61R^ in the ctDNA extracted from 7 melanoma patients with progressive disease who had previously responded to treatment with vemurafenib (*n* = 2) or dabrafenib/trametinib (*n* = 5). Two samples were positive for *NRAS*^Q61K^ and one sample had an *NRAS*^Q61R^ mutation, all three were derived from patients treated with dabrafenib/trametinib.

For all three positive cases the presence of *NRAS* mutations was evaluated in longitudinally collected samples (Figure 3B-3D, Supplementary Figure 2). No mutated *NRAS* was detectable prior to treatment initiation (baseline) or prior to ctDNA rebound. The amount of mutant *NRAS* ctDNA increased as the *BRAF* mutant ctDNA rebounded and both fluctuated in a similar pattern. However, mutant *NRAS* ctDNA was consistently detected at lower quantities than *BRAF* mutant ctDNA. Of note, in patient #17 the escape mutation *NRAS*^Q61K^ could be detected from week 20, however no progressive disease was apparent by radiological scan at week 30 (Figure 3B), once again supporting the value of ctDNA analysis for monitoring melanoma patients undergoing systemic therapies.

## DISCUSSION

Our results demonstrate that quantification of plasma ctDNA is a valuable tool for monitoring tumor dynamics in metastatic melanoma patients. The quantity of ctDNA in plasma tracks the patient's response to treatment and precedes radiological disease progression. Moreover we demonstrate, for the first time, that mutations conferring resistance to BRAF and MEK inhibitors can be detected in the ctDNA of patients undergoing targeted therapies, prior to radiological evidence of progression.

Due to logistics of sample collection and transport, plasma samples used in the study were derived from blood processed 16-24 hours after collection. Although most protocols for the analysis of ctDNA recommend plasma separation within 2-4 hours [31], the agreement of our ctDNA quantities with clinical status indicated that the ctDNA was not compromised in these samples. We have also shown that plasma processing within 4 hours of blood collection did not significantly alter the amount of tumor associated ctDNA relative to processing at 24 hours (Supplementary Figure 3).

We found that low baseline ctDNA (< 10 copies/ml) was predictive of prolonged PFS compared to patients with a high baseline ctDNA, particularly in patients treated with targeted therapies, supporting previous reports [25, 26]. Of note, the predictive value of ctDNA levels on PFS by Cox regression analyses remained significant at various cut-off values from 10-300 copies/ml (data not shown), including 216 copies/ml as described in a recent study by Sanmamed *et al.* [26]. However, all patients with baseline ctDNA < 10 copies per ml responded to therapy and had more than 6 month PFS, thus supporting 10 copies per ml as a biologically significant and data driven threshold, which was not apparent when 216 copies/ml was used as a classifier. Importantly, although a substantial proportion (14/17; 82%) of cases with > 10 ctDNA copies per ml responded to therapy, all rapidly progressing patients (PFS < 6 months) had high ctDNA (> 10 copies/ml) at baseline. Significantly, we found that independent of baseline ctDNA levels, a decrease in ctDNA within 8 weeks after treatment initiation was associated with response to therapy. Thus, monitoring ctDNA early during therapy may provide long-term patient response information that can inform the timing for second line therapy initiation.

Limitations of this study include the small sample size especially of patients treated with immune checkpoint inhibitors and the heterogeneity of the treatments given within this group which are known to have different modes of action and treatment response rates. Future studies are needed to evaluate the predictive value of baseline ctDNA in patients treated with particular immune checkpoint blockade modalities. Moreover, additional studies are required to evaluate the dynamic changes in ctDNA that provide early reliable prediction of treatment response. In particular it will be important to determine whether a decrease in ctDNA after initiation of immunotherapies provides a predictive measure of clinical benefit in patients with a delayed response.

Longitudinal analysis of ctDNA during treatment clearly demonstrated that ctDNA levels closely track clinical disease status. More importantly, a rebound in ctDNA levels correlated with treatment failure, and in at least one case preceded radiological detection of progressive disease. Future studies with more regular collection of plasma samples during therapy will confirm whether rebound of ctDNA generally precedes radiological detection of progression.

One of the most important findings of this study was the identification of acquired resistance via *NRAS* mutations in patients that initially responded to targeted therapy and subsequently progressed. To our knowledge this has not been previously reported for melanoma, and in the setting of tumor tissue heterogeneity, could identify the most important resistant clone to direct further treatment selection. For example in the LOGIC 2 study (clinicaltrials.gov, NCT02159066) where patients are biopsied at progression and a second line treatment selected based on the genetic analysis of the biopsy, results can be confounded by inter- and intra-tumor heterogeneity. We show here that blood monitoring could be utilised to identify the most significant clone at resistance and confirm that it was not detectable at baseline.

Detection of acquired resistance mutations through ctDNA analysis has been previously demonstrated in colorectal cancer with the evolution of mutant KRAS to EGFR blockade [22]. Subsequent studies identified, by whole genome plasma DNA sequencing, the development of resistance to anti-EGFR therapies through KRAS amplification and polysomy of chromosome 12p [32, 33]. Similarly, the resistance–conferring mutation EGFR T790M could be detected in plasma of lung cancer patients following treatment with gefitinib or erlotinib [24, 34]. Altogether these results underscore the utility of ctDNA analysis for non-invasive detection of treatment failure and the development of resistance.

The frequency of patients progressing on BRAF/MEK inhibitors with *NRAS* mutations (43%) was larger than in any other studies, especially for treatment with dabrafenib/trametinib combination [30]. This could reflect the small number of patients analysed, but it is tempting to speculate that *NRAS* mutations were more commonly detected here because ctDNA is derived from the sum of a patient's tumors. Previous studies profiling acquired resistance in melanoma, examined individual progressing lesions, which are known to display intra- and inter-tumoral heterogeneity of resistance mechanisms [11, 12] [35]. Circulating mutant *NRAS* presumably originates from a subset of the melanoma burden, and this is supported by the fact that *NRAS* mutant ctDNA was consistently lower than the *BRAF* mutated ctDNA.

Overall these data confirm that measuring tumor-associated ctDNA is a valuable and simple method to track patient response, tumor evolution and resistance acquisition.

## MATERIALS AND METHODS

### Study design, patient cohorts and ethics statement

Metastatic melanoma patients were enrolled in the study between April 2013 and February 2015, based on having a confirmed *BRAF*^V600E/K/R^ or *NRAS*^Q61R/K/L^ mutation in their melanoma by molecular analysis, at Sir Charles Gairdner Hospital (SCGH) and Fiona Stanley Hospital (FSH) in Perth, Western Australia, and Westmead Hospital and Melanoma Institute Australia, New South Wales, Australia. Written informed consent was obtained from all patients under approved Human Research Ethics Committee protocols from Edith Cowan University (No. 2932), Sir Charles Gairdner Hospital (No. 2007-123) and Royal Prince Alfred Hospital (Protocol No X10-0305 & HREC/10/RPAH/539).

### Patient treatment and follow up

Patients were treated with either vemurafenib, dabrafenib/trametinib combination, ipilimumab, nivolumab, pembrolizumab or ipilimumab/pembrolizumab at the currently approved doses. Patients underwent baseline assessment including medical history, physical examination, and radiologic tumor assessment with computer tomography (CT) or positron emission tomography (PET) scans. Patients were treated at the discretion of their treating oncologist as appropriate for their disease stage, mutational status and Eastern Cooperative Oncology Group (ECOG) performance status. Patients underwent clinical assessment at least monthly, including a physical examination and assessment of biochemical parameters. Tumor responses were assessed radiologically at two to three month intervals. CT scans were assessed by RECIST 1.1 criteria [36] and classified as having a complete response (CR), partial response (PR), stable disease (SD) or progressive disease (PD). Patient information and samples collected are indicated in the Supplementary Table 1.

### Plasma collection and cell free DNA extraction

Patient peripheral blood samples were collected in EDTA vacutainer tubes, stored at 4°C, and processed within 24 hours of being drawn. Blood samples were centrifuged at 1600g for 10 min for plasma collection, followed by a second centrifugation for further plasma clearance. Plasmas were stored at −80°C until extraction. The cell free DNA (cfDNA) was extracted from 1-5 ml of plasma using the QIAamp Circulating Nucleic Acid Kit (Qiagen) according to the manufacturer's instructions. cfDNA was eluted in AVE buffer (Qiagen). DNA samples were stored at −80°C until analysis.

### ctDNA quantification

The ctDNA was quantified by droplet digital PCR. Amplifications were carried out in a 20 μL reaction containing 1× droplet PCR supermix, 250 nM of each probe, 900 nM primers and 5 or 8 μL cfDNA. Samples were analysed for *BRAF*-V600E or V600K mutations depending on the mutation identified in the patient biopsy. The following probes were used: T1799-VIC WT (VIC-CTAGCTACAGTGAAATC-MGBNFQ) and A1799-FAM V600E (6FAM-TAGCTACAGAGAAATC-MGBNFQ), AA1799-1800-FAM V600E2 (6FAM-TAGCTACAGAAAAATC-MGBNFQ) or AA1798-1799-FAM V600K (6FAM-TAGCTACAAAGAAATC-MGBNFQ). The following primers were used for both assays: 5′-CTACTGTTTTCCTTTACTTACTACTACACCTCAGA-3′ (forward) and 5′-ATCCAGACAACTGTTCAAACTGATG-3′ (reverse). Probes and primers were custom synthesised by Life Technologies. *NRAS* mutations Q61K and Q61R were tested using the commercial PrimePCR mutation assays (BioRad).

Droplets were generated and analysed using the QX200 system (Bio-Rad). Amplifications were performed using the following conditions: 1 cycle of 95°C for 10 minutes, 40 cycles of 94°C for 30 seconds and 55°C for 1 minute, and 1 cycle of 98°C for 10 minutes. A positive control, a healthy control and a no template control were included in each run. QuantaSoft version 1.6.6 analysis software (Bio-Rad) was used for data acquisition and analysis. Only tests providing more than 10,000 droplets were used for analysis. The number of copies of mutated DNA per 20 μl reaction was extrapolated to calculate copies per ml using the following equation:

Copies/ml of plasma=C*EV/TV/PV.

PV = Volume of plasma used for cfDNA extraction (ml)
EV = Volume in which cfDNA was eluted (μl)
TV = Volume of cfDNA added to the PCR reaction (μl)
C = copies/20μl (data derived from QuantaSoft)

To facilitate graphical representation and statistical analysis, samples with no detectable ctDNA were given a value of 1 copy per ml. Control samples derived from plasma of age matched healthy individuals were used to determine the specificity of each assay (Supplementary Table 2). Most samples were tested only once against each mutation due to the limited amount of cfDNA available for analysis. We showed in a subset of 20 samples the reproducibility of the assay (Supplementary Figure 4).

### Statistical analysis

PFS was defined as the time from the date of initiating therapy to the date of first reported PD or censored at the most recent visit. Baseline ctDNA was tested for association with overall response and 6-month PFS using one-sided Mann-Whitney U test.

The ctDNA data was dichotomised in order to determine the best cut-off value to discriminate between groups of responders or non-responders, as well as those with PFS of more or less than 6 months (6mo PFS). Comparisons were performed using Fisher's exact tests. The reported p-values have not been adjusted for multiple comparisons.

Univariate Cox proportional hazards regression analyses were performed to examine association of ctDNA levels, age, sex, metastatic disease stage and LDH status with PFS. Multivariate Cox regression models were evaluated using a stepwise approach with bidirectional elimination to determine the best fit model. Results were analysed in SPSS v22.0 and GraphPad Prism 5. Results were considered statistically significant at *p* < 0.05.

## SUPPLEMENTARY MATERIAL FIGURES AND TABLES



## References

[1] Davies H, Bignell GR, Cox C, Stephens P, Edkins S, Clegg S, Teague J, Woffendin H, Garnett MJ, Bottomley W, Davis N, Dicks E, Ewing R, Floyd Y, Gray K, Hall S (2002). Mutations of the BRAF gene in human cancer. Nature.

[2] Hodis E, Watson IR, Kryukov GV, Arold ST, Imielinski M, Theurillat JP, Nickerson E, Auclair D, Li L, Place C, Dicara D, Ramos AH, Lawrence MS, Cibulskis K, Sivachenko A, Voet D (2012). A landscape of driver mutations in melanoma. Cell.

[3] Rubinstein JC, Sznol M, Pavlick AC, Ariyan S, Cheng E, Bacchiocchi A, Kluger HM, Narayan D, Halaban R (2010). Incidence of the V600K mutation among melanoma patients with BRAF mutations, and potential therapeutic response to the specific BRAF inhibitor PLX4032. J Transl Med.

[4] Lovly CM, Dahlman KB, Fohn LE, Su Z, Dias-Santagata D, Hicks DJ, Hucks D, Berry E, Terry C, Duke M, Su Y, Sobolik-Delmaire T, Richmond A, Kelley MC, Vnencak-Jones CL, Iafrate AJ (2012). Routine multiplex mutational profiling of melanomas enables enrollment in genotype-driven therapeutic trials. PLoS One.

[5] Klein O, Clements A, Menzies AM, O'Toole S, Kefford RF, Long GV (2013). BRAF inhibitor activity in V600R metastatic melanoma. European journal of cancer.

[6] Menzies AM, Haydu LE, Visintin L, Carlino MS, Howle JR, Thompson JF, Kefford RF, Scolyer RA, Long GV (2012). Distinguishing clinicopathologic features of patients with V600E and V600K BRAF-mutant metastatic melanoma. Clinical cancer research.

[7] Chapman PB, Hauschild A, Robert C, Haanen JB, Ascierto P, Larkin J, Dummer R, Garbe C, Testori A, Maio M, Hogg D, Lorigan P, Lebbe C, Jouary T, Schadendorf D, Ribas A (2011). Improved survival with vemurafenib in melanoma with BRAF V600E mutation. The New England journal of medicine.

[8] Hauschild A, Grob JJ, Demidov LV, Jouary T, Gutzmer R, Millward M, Rutkowski P, Blank CU, Miller WH, Kaempgen E, Martin-Algarra S, Karaszewska B, Mauch C, Chiarion-Sileni V, Martin AM, Swann S (2012). Dabrafenib in BRAF-mutated metastatic melanoma: a multicentre, open-label, phase 3 randomised controlled trial. Lancet.

[9] Long GV, Stroyakovskiy D, Gogas H, Levchenko E, de Braud F, Larkin J, Garbe C, Jouary T, Hauschild A, Grob JJ, Chiarion Sileni V, Lebbe C, Mandala M, Millward M, Arance A, Bondarenko I (2014). Combined BRAF and MEK inhibition versus BRAF inhibition alone in melanoma. The New England journal of medicine.

[10] Robert C, Karaszewska B, Schachter J, Rutkowski P, Mackiewicz A, Stroiakovski D, Lichinitser M, Dummer R, Grange F, Mortier L, Chiarion-Sileni V, Drucis K, Krajsova I, Hauschild A, Lorigan P, Wolter P (2015). Improved overall survival in melanoma with combined dabrafenib and trametinib. The New England journal of medicine.

[11] Shi H, Hugo W, Kong X, Hong A, Koya RC, Moriceau G, Chodon T, Guo R, Johnson DB, Dahlman KB, Kelley MC, Kefford RF, Chmielowski B, Glaspy JA, Sosman JA, van Baren N (2014). Acquired resistance and clonal evolution in melanoma during BRAF inhibitor therapy. Cancer Discov.

[12] Rizos H, Menzies AM, Pupo GM, Carlino MS, Fung C, Hyman J, Haydu LE, Mijatov B, Becker TM, Boyd SC, Howle J, Saw R, Thompson JF, Kefford RF, Scolyer RA, Long GV (2014). BRAF inhibitor resistance mechanisms in metastatic melanoma: spectrum and clinical impact. Clinical cancer research.

[13] Long GV, Fung C, Menzies AM, Pupo GM, Carlino MS, Hyman J, Shahheydari H, Tembe V, Thompson JF, Saw RP, Howle J, Hayward NK, Johansson P, Scolyer RA, Kefford RF, Rizos H (2014). Increased MAPK reactivation in early resistance to dabrafenib/trametinib combination therapy of BRAF-mutant metastatic melanoma. Nature communications.

[14] Hamid O, Robert C, Daud A, Hodi FS, Hwu WJ, Kefford R, Wolchok JD, Hersey P, Joseph RW, Weber JS, Dronca R, Gangadhar TC, Patnaik A, Zarour H, Joshua AM, Gergich K (2013). Safety and tumor responses with lambrolizumab (anti-PD-1) in melanoma. The New England journal of medicine.

[15] Hodi FS, O'Day SJ, McDermott DF, Weber RW, Sosman JA, Haanen JB, Gonzalez R, Robert C, Schadendorf D, Hassel JC, Akerley W, van den Eertwegh AJ, Lutzky J, Lorigan P, Vaubel JM, Linette GP (2010). Improved survival with ipilimumab in patients with metastatic melanoma. The New England journal of medicine.

[16] Robert C, Long GV, Brady B, Dutriaux C, Maio M, Mortier L, Hassel JC, Rutkowski P, McNeil C, Kalinka-Warzocha E, Savage KJ, Hernberg MM, Lebbe C, Charles J, Mihalcioiu C, Chiarion-Sileni V (2015). Nivolumab in previously untreated melanoma without BRAF mutation. The New England journal of medicine.

[17] Robert C, Ribas A, Wolchok JD, Hodi FS, Hamid O, Kefford R, Weber JS, Joshua AM, Hwu WJ, Gangadhar TC, Patnaik A, Dronca R, Zarour H, Joseph RW, Boasberg P, Chmielowski B (2014). Anti-programmed-death-receptor-1 treatment with pembrolizumab in ipilimumab-refractory advanced melanoma: a randomised dose-comparison cohort of a phase 1 trial. Lancet.

[18] Wolchok JD, Hoos A, O'Day S, Weber JS, Hamid O, Lebbe C, Maio M, Binder M, Bohnsack O, Nichol G, Humphrey R, Hodi FS (2009). Guidelines for the evaluation of immune therapy activity in solid tumors: immune-related response criteria. Clinical cancer research.

[19] Nishino M, Giobbie-Hurder A, Gargano M, Suda M, Ramaiya NH, Hodi FS (2013). Developing a common language for tumor response to immunotherapy: immune-related response criteria using unidimensional measurements. Clinical cancer research.

[20] Bettegowda C, Sausen M, Leary RJ, Kinde I, Wang Y, Agrawal N, Bartlett BR, Wang H, Luber B, Alani RM, Antonarakis ES, Azad NS, Bardelli A, Brem H, Cameron JL, Lee CC (2014). Detection of circulating tumor DNA in early- and late-stage human malignancies. Sci Transl Med.

[21] Dawson SJ, Tsui DW, Murtaza M, Biggs H, Rueda OM, Chin SF, Dunning MJ, Gale D, Forshew T, Mahler-Araujo B, Rajan S, Humphray S, Becq J, Halsall D, Wallis M, Bentley D (2013). Analysis of circulating tumor DNA to monitor metastatic breast cancer. The New England journal of medicine.

[22] Diaz LA, Williams RT, Wu J, Kinde I, Hecht JR, Berlin J, Allen B, Bozic I, Reiter JG, Nowak MA, Kinzler KW, Oliner KS, Vogelstein B (2012). The molecular evolution of acquired resistance to targeted EGFR blockade in colorectal cancers. Nature.

[23] Heitzer E, Ulz P, Geigl JB (2015). Circulating tumor DNA as a liquid biopsy for cancer. Clin Chem.

[24] Murtaza M, Dawson SJ, Tsui DW, Gale D, Forshew T, Piskorz AM, Parkinson C, Chin SF, Kingsbury Z, Wong AS, Marass F, Humphray S, Hadfield J, Bentley D, Chin TM, Brenton JD (2013). Non-invasive analysis of acquired resistance to cancer therapy by sequencing of plasma DNA. Nature.

[25] Ascierto PA, Minor D, Ribas A, Lebbe C, O'Hagan A, Arya N, Guckert M, Schadendorf D, Kefford RF, Grob JJ, Hamid O, Amaravadi R, Simeone E, Wilhelm T, Kim KB, Long GV (2013). Phase II trial (BREAK-2) of the BRAF inhibitor dabrafenib (GSK2118436) in patients with metastatic melanoma. Journal of clinical oncology.

[26] Sanmamed MF, Fernandez-Landazuri S, Rodriguez C, Zarate R, Lozano MD, Zubiri L, Perez-Gracia JL, Martin-Algarra S, Gonzalez A (2015). Quantitative cell-free circulating BRAFV600E mutation analysis by use of droplet digital PCR in the follow-up of patients with melanoma being treated with BRAF inhibitors. Clin Chem.

[27] Lipson EJ, Velculescu VE, Pritchard TS, Sausen M, Pardoll DM, Topalian SL, Diaz LA (2014). Circulating tumor DNA analysis as a real-time method for monitoring tumor burden in melanoma patients undergoing treatment with immune checkpoint blockade. J Immunother Cancer.

[28] Tsao SC, Weiss J, Hudson C, Christophi C, Cebon J, Behren A, Dobrovic A (2015). Monitoring response to therapy in melanoma by quantifying circulating tumour DNA with droplet digital PCR for BRAF and NRAS mutations. Scientific reports.

[29] Hodi FS, Ribas A, Daud A, Hamid O, Robert C, Kefford R (2014). Evaluation of Immune-Related Response Criteria (irRC) in Patients With Advanced Melanoma Treated With the Anti-PD-1 Monoclonal Antibody Pembrolizumab (MK-3475).

[30] Long GV, Atkinson V, Ascierto P, Brady B (2014). Nivolumab Improved Survival vs Dacarbazine in Patients with Untreated Advanced Melanoma.

[31] Xue X, Teare MD, Holen I, Zhu YM, Woll PJ (2009). Optimizing the yield and utility of circulating cell-free DNA from plasma and serum. Clin Chim Acta.

[32] Heitzer E, Auer M, Hoffmann EM, Pichler M, Gasch C, Ulz P, Lax S, Waldispuehl-Geigl J, Mauermann O, Mohan S, Pristauz G, Lackner C, Hofler G, Eisner F, Petru E, Sill H (2013). Establishment of tumor-specific copy number alterations from plasma DNA of patients with cancer. Int J Cancer.

[33] Mohan S, Heitzer E, Ulz P, Lafer I, Lax S, Auer M, Pichler M, Gerger A, Eisner F, Hoefler G, Bauernhofer T, Geigl JB, Speicher MR (2014). Changes in colorectal carcinoma genomes under anti-EGFR therapy identified by whole-genome plasma DNA sequencing. PLoS Genet.

[34] Oxnard GR, Paweletz CP, Kuang Y, Mach SL, O'Connell A, Messineo MM, Luke JJ, Butaney M, Kirschmeier P, Jackman DM, Janne PA (2014). Noninvasive detection of response and resistance in EGFR-mutant lung cancer using quantitative next-generation genotyping of cell-free plasma DNA. Clinical cancer research.

[35] Wilmott JS, Tembe V, Howle JR, Sharma R, Thompson JF, Rizos H, Lo RS, Kefford RF, Scolyer RA, Long GV (2012). Intratumoral molecular heterogeneity in a BRAF-mutant, BRAF inhibitor-resistant melanoma: a case illustrating the challenges for personalized medicine. Molecular cancer therapeutics.

[36] Eisenhauer EA, Therasse P, Bogaerts J, Schwartz LH, Sargent D, Ford R, Dancey J, Arbuck S, Gwyther S, Mooney M, Rubinstein L, Shankar L, Dodd L, Kaplan R, Lacombe D, Verweij J (2009). New response evaluation criteria in solid tumours: revised RECIST guideline (version 1.1). European journal of cancer.

