# Supplementary material for: Circulating tumor DNA to monitor treatment response and detect acquired resistance in patients with metastatic melanoma
Source: Oncotarget. 2015 Sep 22;6(39):42008–18. doi: 10.18632/oncotarget.5788 (PMC4747205; doi:10.18632/oncotarget.5788)
Supplement: Supplementary file 1 [file oncotarget-06-42008-s001.pdf]

Circulating tumor DNA to monitor treatment response and detect acquired resistance in patients with metastatic melanoma

SUPPLEMENTARY MATERIAL

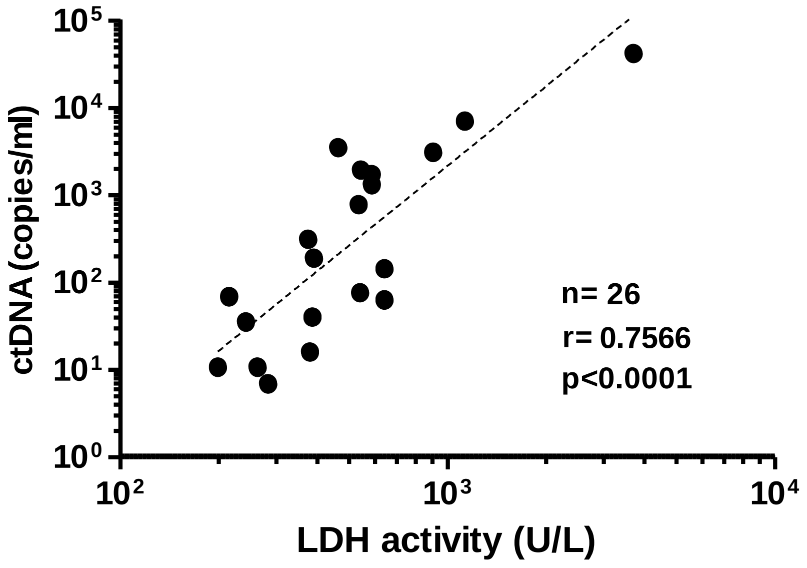

Supplementary Figure 1: Correlation of baseline ctDNA concentration and LDH activity (n=26). The correlation of baseline ctDNA levels and LDH activity was assessed using Spearman's rank coefficient. Due to differences between the methods employed to measure LDH by the clinical laboratories, only data from one of the sites was used for the correlation analysis.

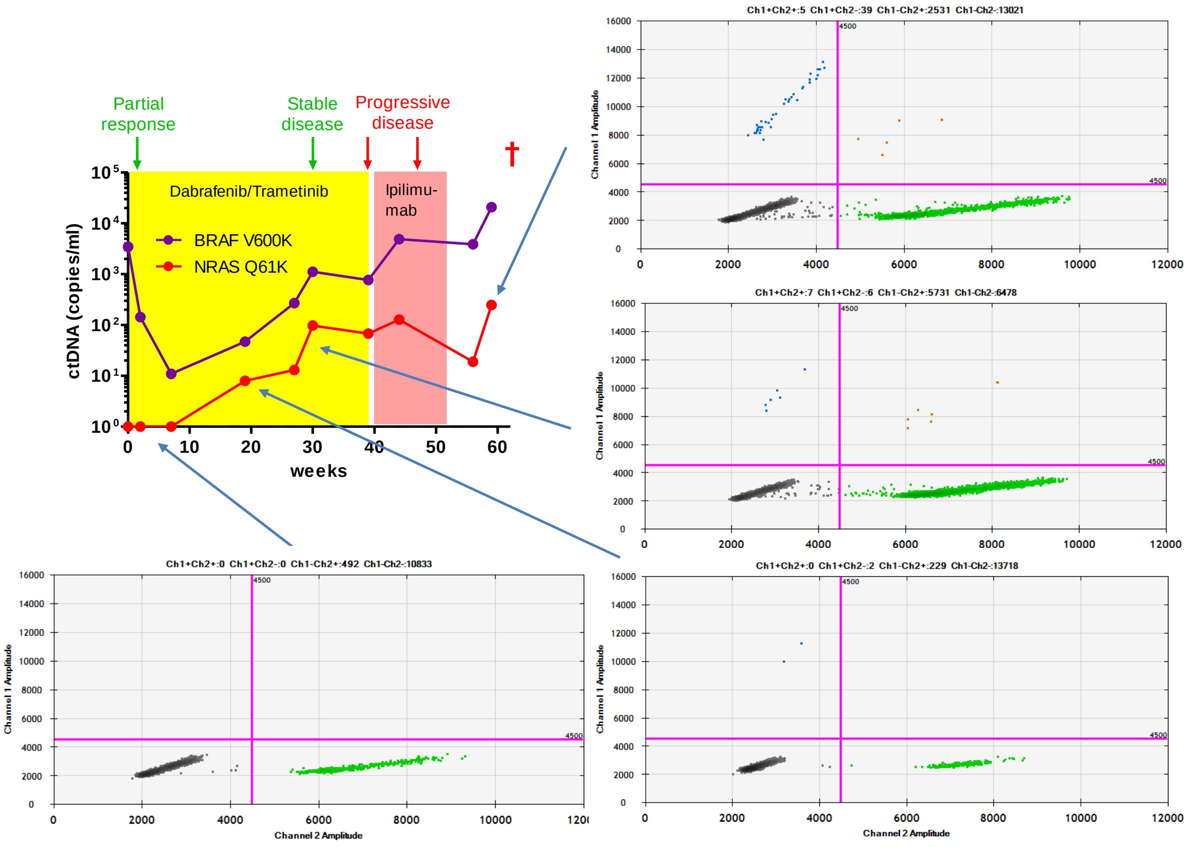

Supplementary Figure 2: Levels of mutant *BRAF* and *NRAS* ctDNA in plasma collected longitudinally from patient #17 during treatment with dabrafenib and trametinib combination therapy, followed by ipilimumab treatment. Clinical outcomes revealed by CT scans are indicated at each assessment time. Patient's death is indicated by a red cross (†). 2D plots of *NRAS* Q61K assay of four time points during the course of treatment are displayed, with droplets containing WT DNA in green, *NRAS* Q61K in blue and double negative in black.

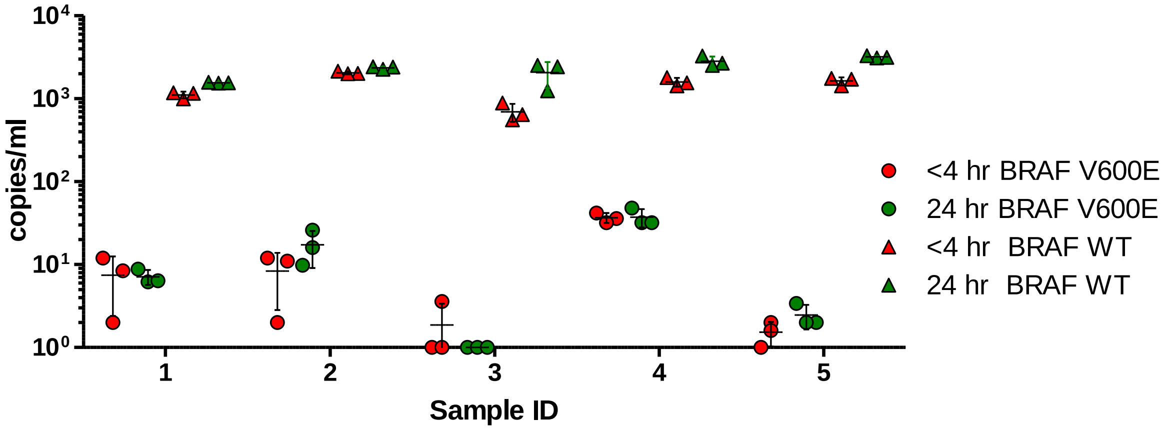

	<4 hr BRAF V600E			24 hr BRAF V600E			<4 hr BRAF WT			24 hr BRAF WT		
	Y1	Y2	Y3	Y1	Y2	Y3	Y1	Y2	Y3	Y1	Y2	Y3
T1	8.4	12.0	2.0	6.2	8.8	6.4	1160.0	1180.0	996.0	1540.0	1560.0	1580.0
T2	2.0	12.0	11.0	26.0	9.8	16.0	2140.0	2020.0	2000.0	2420.0	2400.0	2260.0
T3	3.6	1.0	1.0	1.0	1.0	1.0	886.0	558.0	642.0	2420.0	1240.0	2520.0
T4	36.0	42.0	32.0	48.0	32.0	32.0	1800.0	1552.0	1424.0	3280.0	2680.0	2520.0
T5	2.0	1.6	1.0	2.0	3.4	2.0	1720.0	1760.0	1424.0	3100.0	3300.0	3160.0

Supplementary Figure 3: Comparison of ctDNA quantification relative to plasma collection time. Plasmas from 5 blood samples were separated at either 2-4 hours after being drawn or bloods were left at 4˚C for 24 hours and then plasma was collected. All plasmas were stored at -80˚C until processing. cfDNA was isolated from plasmas and tested by ddPCR for V600E mutations in triplicate. The copies of mutant and wild-type (WT) DNA were calculated for each sample. Samples with undetectable levels of DNA were given a value of 1 copy/ml. No significant difference was observed between mutant DNA copies detected at <4 and 24 hours in a paired t-test ($p>0.05$). Of note, the amount of WT DNA significantly increases in plasma if collected 24 hours after blood draw ($p=0.0178$), possibly due to the lysis of white blood cells during this period.

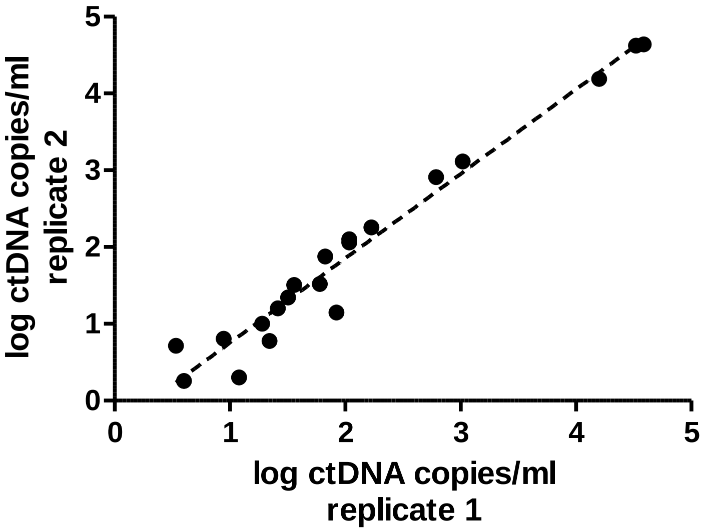

Supplementary Figure 4: Reproducibility of droplet digital PCR. Data from 20 samples that were run in duplicate with either the *BRAF* V600E or the *BRAF* V600K assay. Replicates were run in separate experiments. R^2 = 0.96 with a standard deviation of residuals of 0.26.

Supplementary Table 1: Characteristics of patients in the study

Case	Age	Sex	Mutation	Stage	Treatment	Treatment type (T-Targeted or I-Immunotherapy	LDH (U/L)	Elevated LDH	Baseline ctDNA copies/ml	Response (Y/N)	PFS-6mo (26w)	ctDNA copies/ml at 4-8 weeks follow up	Number of plasma tested in longitudinal follow up
1	35	F	BRAF V600E	M1c	Dabrafenib	T	335	1	undetectable	Y	Y	not done	-
2	57	M	BRAF V600E	M1c	Dabrafenib/Trametenib	T	215	0	67.5	Y	Y	0	-
3	62	M	BRAF V600E	M1c	Dabrafenib/Trametenib	T	not available	not available	620.0	Y	Y	not available	-
4	40	M	BRAF V600E	M1a	Dabrafenib/Trametenib	T	151	0	undetectable	Y	Y	not done	-
5	59	F	BRAF V600E	M1c	Dabrafenib/Trametenib	T	178	0	undetectable	Y	Y	not done	-
7	62	M	BRAF V600E	M1c	Dabrafenib/Trametenib	T	586	0	1695.0	N	N	not available	-
8	31	M	BRAF V600E	M1c	Dabrafenib/Trametenib	T	199	0	undetectable	Y	Y	not done	-
9	75	M	BRAF V600E	M1c	Dabrafenib/Trametenib	T	203	0	undetectable	Y	Y	not done	-
10	60	M	BRAF V600E	M1c	Dabrafenib/Trametenib	T	283	0	6.8	Y	Y	not available	-
11	67	M	BRAF V600K	M1c	Dabrafenib/Trametenib	T	3700	1	41400.0	Y	Y	6	2
12	57	M	BRAF V600E	M1c	Dabrafenib/Trametenib	T	403	0	undetectable	Y	Y	not done	-
13	66	F	BRAF V600K	M1c	Dabrafenib/Trametenib	T	387	0	39.8	Y	Y	not available	-
14	31	M	BRAF V600E	M1c	Dabrafenib/Trametenib	T	586	0	1297.5	Y	N	0	8
15	59	M	BRAF V600E	M1c	Dabrafenib/Trametenib	T	213	0	undetectable	Y	Y	not done	-
16	53	M	BRAF V600E	M1c	Dabrafenib/Trametenib	T	543	0	1912.5	Y	N	0	5
17	44	F	BRAF V600K	M1c	Dabrafenib/Trametenib	T	464	0	3472.5	Y	Y	11	9
18	59	F	BRAF V600K	M1c	Dabrafenib/Trametenib	T	1130	1	6975.0	Y	N	0	6
19	72	F	BRAF V600E	M1c	Dabrafenib/Trametenib	T	206	0	undetectable	Y	Y	not done	-
20	45	M	BRAF V600E	M1c	Dabrafenib/Trametenib	T	540	0	75.0	N	N	112.5	-
21	45	F	BRAF V600E	M1c	Dabrafenib/Trametenib	T	244	0	165.0	Y	Y	not available	-
22	20	M	BRAF V600E	M1c	Dabrafenib/Trametenib	T	not elevated	0	27.5	Y	N	not available	-
23	43	M	BRAF V600E	M1c	Dabrafenib/Trametenib	T	132	0	undetectable	Y	Y	not done	-
24	59	F	BRAF V600E	M1c	Dabrafenib/Trametenib	T	187	0	undetectable	Y	Y	not done	-
25	71	M	BRAF V600E	M1b	Dabrafenib/Trametenib	T	203	0	152.5	Y	N	not available	-
26	75	M	BRAF V600E	M1b	Dabrafenib/Trametenib	T	219	0	15.0	Y	Y	not available	-
27	30	M	BRAF V600E	M1c	Vemurafenib	T	263	0	10.5	Y	N	0	2
28	43	M	BRAF V600E	M1c	Vemurafenib	T	904	1	3060.0	N	Y	0	-
29	83	F	BRAF V600E	M1a	Vemurafenib	T	not available	not available	undetectable	Y	Y	not done	-
30	61	F	BRAF V600E	M1c	Vemurafenib	T	199	0	10.5	Y	Y	0	6
31	61	F	BRAF V600K	M1c	Ipilimumab	I	391	0	187.5	Y	Y	22.5	4
32	77	M	BRAF V600E	M1a	Ipilimumab	I	242	0	34.5	N	N	592.5	-
33	53	M	BRAF V600E	M1c	Ipilimumab	I	642	1	142.5	N	N	1822.5	-
34	59	F	BRAF V600K	M1c	Ipilimumab	I	380	0	15.8	N	N	27	-
35	44	F	BRAF V600K	M1c	Ipilimumab	I	535	0	765.0	N	N	6300	-
36	48	F	BRAF V600R	M1c	Ipilimumab	I	641	1	62.0	N	N	916	-
37	35	M	BRAF V600E	M1c	Ipilimumab	I	206	0	12.2	N	N	60	-
38	51	M	NRAS Q61L	M1a	Ipilimumab	I	not available	not available	10.0	N	N	150	-
39	31	M	BRAF V600E	M1c	Ipilimumab	I	375	0	307.5	N	N	97.5	-
40	33	M	BRAF V600E	M1c	Ipilimumab/Pembrolizumab	I	257	1	1607.5	Y	Y	2.5	-
41	46	M	NRAS Q61R	M1b	Nivolumab	I	not elevated	0	5.0	Y	Y	12.5	-
42	64	M	NRAS Q61R	M1c	Nivolumab	I	not elevated	0	112.5	N	N	0	-
43	87	M	BRAF V600K	M1c	Nivolumab	I	246	0	undetectable	Y	Y	not done	-
44	77	M	BRAF V600E	M1a	Pembrolizumab	I	467	0	undetectable	Y	Y	not done	-
45	66	M	BRAF V600R	M1c	Pembrolizumab	I	227	0	1.6	Y	Y	not available	-
46	69	F	BRAF V600E	M1a	Pembrolizumab	I	246	0	16.0	Y	Y	10	-
47	31	M	BRAF V600E	M1c	Pembrolizumab	I	not available	not available	45.0	N	N	14	-
48	69	M	NRAS Q61K	M1b	Pembrolizumab	I	315	1	357.5	N	N	282.5	-
49	37	M	BRAF V600E	M1c	Pembrolizumab	I	573	1	57302.5	N	N	11675	-

Supplementary Table 2: Sensitivity and specificity of ddPCR assay for V600E

ctDNA	Melanoma Patients		Healthy controls		χ^2 p-value	Sensitivity	Specificity
	Positive	Negative	Positive	Negative			
***BRAF* V600E**	22	12	0	22	>0.001	64.7%	100%
***BRAF* V600K**	7	1	0	23	>0.001	87.5%	100%
***BRAF* V600R**	2	0	0	10			100%
***NRAS* Q61R**	2	0	2*	11			85%
***NRAS* Q61K**	1	0	0	19			100%
***NRAS* Q61L**	1	0	3[#]	9			75%

* ≤7 copies/ml of NRAS Q61R detected in 2 healthy controls
[#] ≤7 copies/ml of NRAS Q61L detected in 3 healthy controls
No statistical analysis was done for mutations tested in 1 or 2 melanoma patients.
